# Supplementary material for: Electronic Properties of Tetraazaperopyrene Derivatives on Au(111): Energy-Level Alignment and Interfacial Band Formation
Source: J Phys Chem C Nanomater Interfaces. 2021 Sep 2;125(36):19969–79. doi: 10.1021/acs.jpcc.1c04217 (PMC8450938; doi:10.1021/acs.jpcc.1c04217)
Supplement: Supplementary file 1 — jp1c04217_si_001.pdf [file jp1c04217_si_001.pdf]

Supporting Information to:

Electronic Properties of Tetraazaperopyrene Derivatives on Au(111): Energy Level Alignment and Interfacial Band Formation

Arnulf Stein,[1] Daniela Rolf,[2] Christian Lotze,[2] Sascha Feldmann,[1] David Gerbert,[1] Benjamin Günther,[3] Andreas Jeindl,[4] Johannes J. Cartus,[4] Oliver T. Hofmann,[4] Lutz H. Gade,[3] Katharina J. Franke,[2] and Petra Tegeder[1]

[1]*Physikalisch-Chemisches Institut, Universität Heidelberg, Im Neuenheimer Feld 253, 69120 Heidelberg, Germany*

[2]*Fachbereich Physik, Freie Universität Berlin, Arnimallee 14, D-14195 Berlin, Germany*

[3]*Anorganisch-Chemisches Institut, Universität Heidelberg, Im Neuenheimer Feld 270, 69120 Heidelberg, Germany*

[4]*Technische Universität Graz, Institut für Festkörperphysik, NAWI Graz, Petersgasse 16, 8010 Graz, Austria*

CONTENTS

S1. Temperature-programmed desorption data S3

S2. Mapping molecular interactions of TAPP-CF_3 S4

S3. Calculated geometry and Mulliken charge of TAPP-CF_3 on Au(111) S4

S4. Two-photon photoemission data of TAPP-H/Au(111) and TAPP-C_3F_7/Au(111) S5

S5. Adsorption properties of TAPP-C_3F_7/Au(111) S7

S1. TEMPERATURE-PROGRAMMED DESORPTION DATA

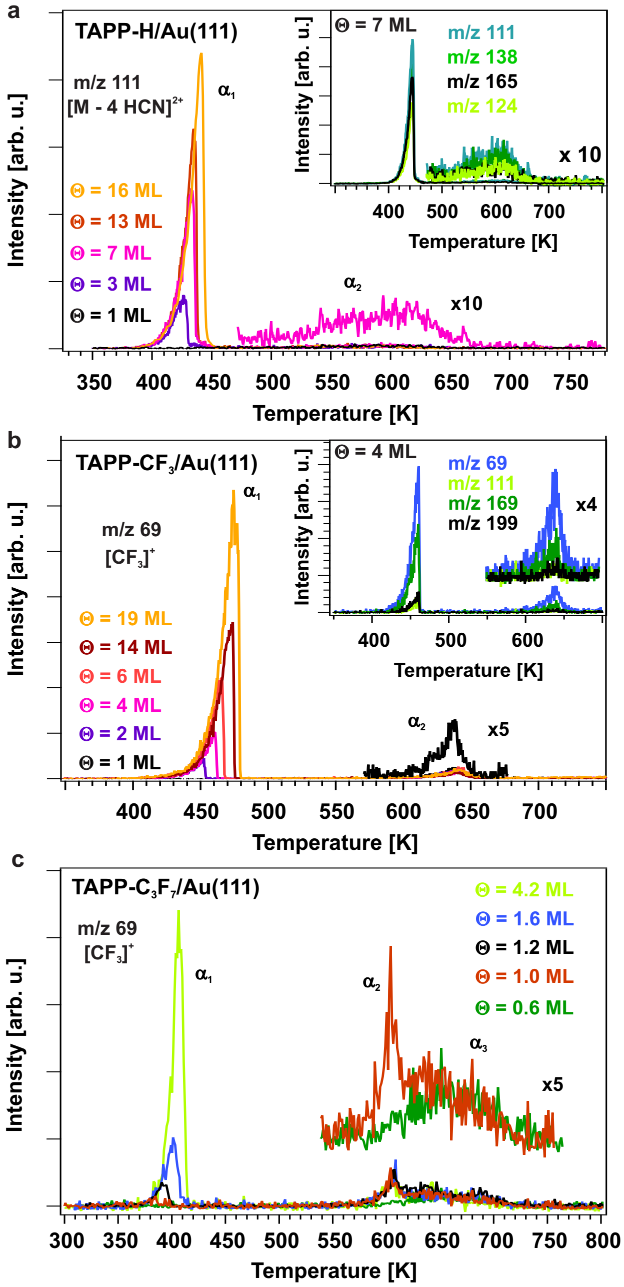

FIG. S1. TPD spectra of a) TAPP-H/Au(111), b) TAPP-CF_3/Au(111), and c) TAPP-C_3F_7/Au(111) with different initial coverages recorded with a heating rate of β = 1 K/s. The insets in a) and b) display TPD spectra recorded at an initial coverage of 7 and 4 ML for different fragment masses.

As can be seen from the temperature-programmed desorption (TPD) spectra (Figure S1)

multilayer desorption (labeled as α_1) takes place between 400 and 470 K, depending on the TAPP derivative and the initial coverage, while monolayer desorption (labeled as α_2) occurs at temperatures above 520 K. Thus, to prepare a defined monolayer (ML) coverage deposition of a multilayer coverage followed by annealing to 500 K to desorb the multilayer has been applied.

S2. MAPPING MOLECULAR INTERACTIONS OF TAPP-CF_3

To verify the structural model we mapped the pairwise energies of a flat-lying dimer in gas phase. Figure S2 shows the interactions of the dimer with the first molecule in the center and the second one moved around the first such that one of the dots is in the center of the second molecule. The color of this dot then indicates the interaction energy of the dimer. It shows that the structure proposed (see main manuscript Fig. 2d) is energetically very beneficial

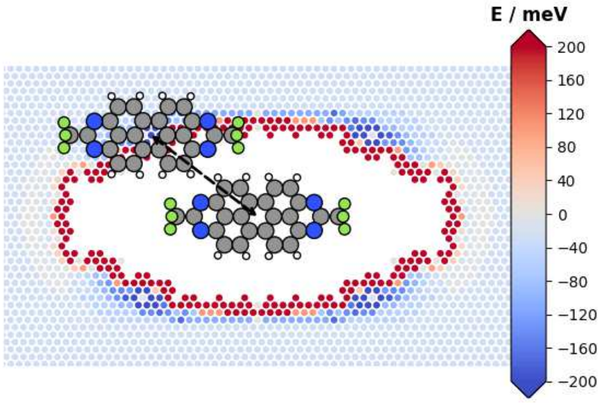

FIG. S2. Pairwise interactions of TAPP-CF_3

S3. CALCULATED GEOMETRY AND MULLIKEN CHARGE OF TAPP-CF_3 ON AU(111)

Figure S3 show a side view of the DFT calculated geometry of TAPP-CF_3 on Au(111), indicating a bending of the CF_3-groups. In addition, the calculated Mulliken charges are displayed.

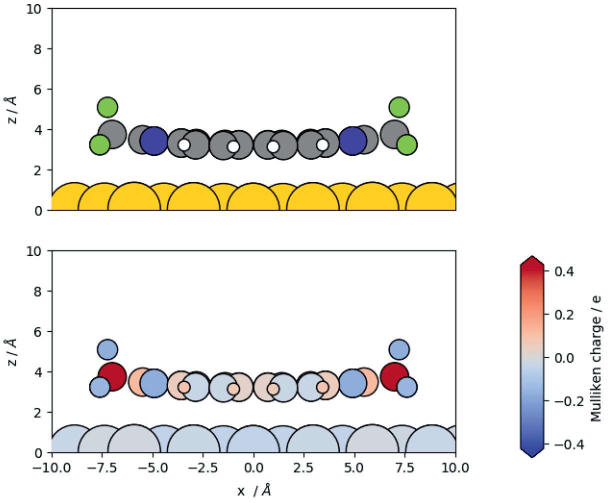

FIG. S3. Side view of the geometry of TAPP-CF_3 on Au(111). The top view shows the system with gold atoms colored yellow, carbon grey, nitrogen blue and fluorine red. The bottom view shows each atom colored according to its Mulliken charge. The overall charge of the TAPP-CF_3 molecule amounts to 0.2 electrons.

S4. TWO-PHOTON PHOTOEMISSION DATA OF TAPP-H/AU(111) AND TAPP-C_3F_7/AU(111)

Figure S4 shows a set of 2PPE measurements carried out at the TAPP-H/Au(111) interface, with a coverage of 1 ML TAPP-H adsorbed on the gold surface. The spectra can be regarded as a sum of an exponential secondary electron background and peaks resulting from occupied or unoccupied electronic states. The observed peaks are highlighted by dashed lines. In overall four unoccupied molecular orbitals (UMO) including the LUMO, the highest occupied molecular orbital (HOMO), an occupied interface hybrid band (OIHB), two d-band features, the shifted surface state (SS') and the first and second image potential state (IPS) are observed. The assignment is based on the photon energy dependent peak position. Calculating the energies of the electronic states in respect to E_{Fermi} results in $E_{SS'} = -0.31 \pm 0.11eV$, $E_{HOMO} = -1.73 \pm 0.06eV$, $E_{OIHB} = -2.48 \pm 0.06eV$, $E_{LUMO} = 1.36 \pm 0.06eV$, $E_{UMO_1} = 1.53 \pm 0.10eV$, $E_{UMO_2} = 1.96 \pm 0.08eV$, $E_{IPS_{n=1}} = 3.94 \pm 0.09eV$, $E_{IPS_{n=2}} = 4.50 \pm 0.11eV$, and $E_{UMO_5} = 5.21 \pm 0.06eV$.

Figure S5 shows a set of 2PPE measurements carried out at the TAPP-C_3F_7/Au(111) interface, with a coverage of 1 ML TAPP-C_3F_7 adsorbed on the gold surface. The spectra can

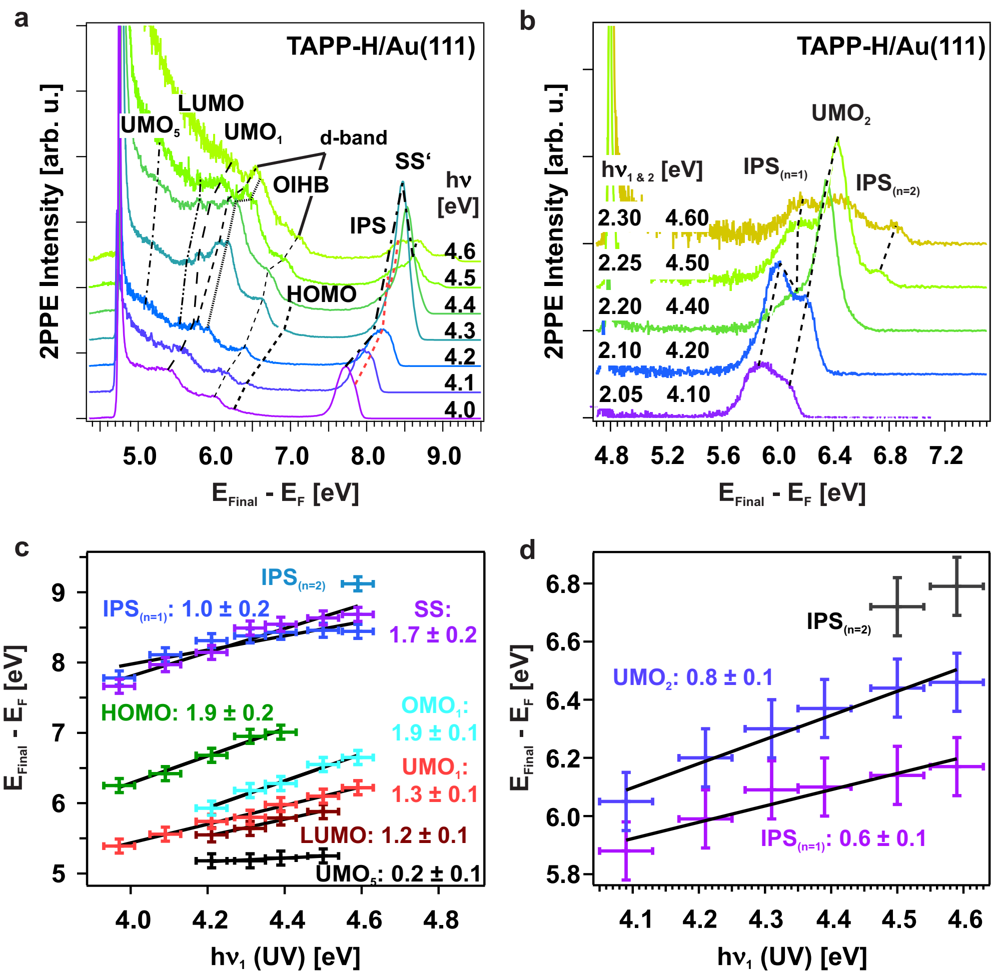

FIG. S4. Photon-energy dependent 2PPE measurements at the TAPP-H/Au(111) interface. a) One-color and b) two-color 2PPE spectra. The peak positions are highlighted by dashed lines. c) and d) Photon-energy-dependent peak positions to assign the observed photoemission signals to occupied, unoccupied intermediate or final electronic states. A slope of 1 suggests that a peak originates from an unoccupied intermediate state, a slope of zero from an unoccupied final state (located above the vacuum level), while a slope of 2 is related to peaks originating from occupied states.

be regarded as a sum of an exponential secondary electron background and peaks resulting from occupied or unoccupied electronic states. The observed peaks are highlighted by dashed lines. In overall two UMOs, an unoccupied interface hybrid band (UIHB), an OIHB, the HOMO, three d-band features, the SS' and the first IPS are observed. The assignment is based on the photon energy dependent peak position. Calculating the energies of the electronic states in respect to E_{Fermi} results in $E_{SS'} = -0.29 \pm 0.05\ eV$, $E_{HOMO} = -1.73 \pm$

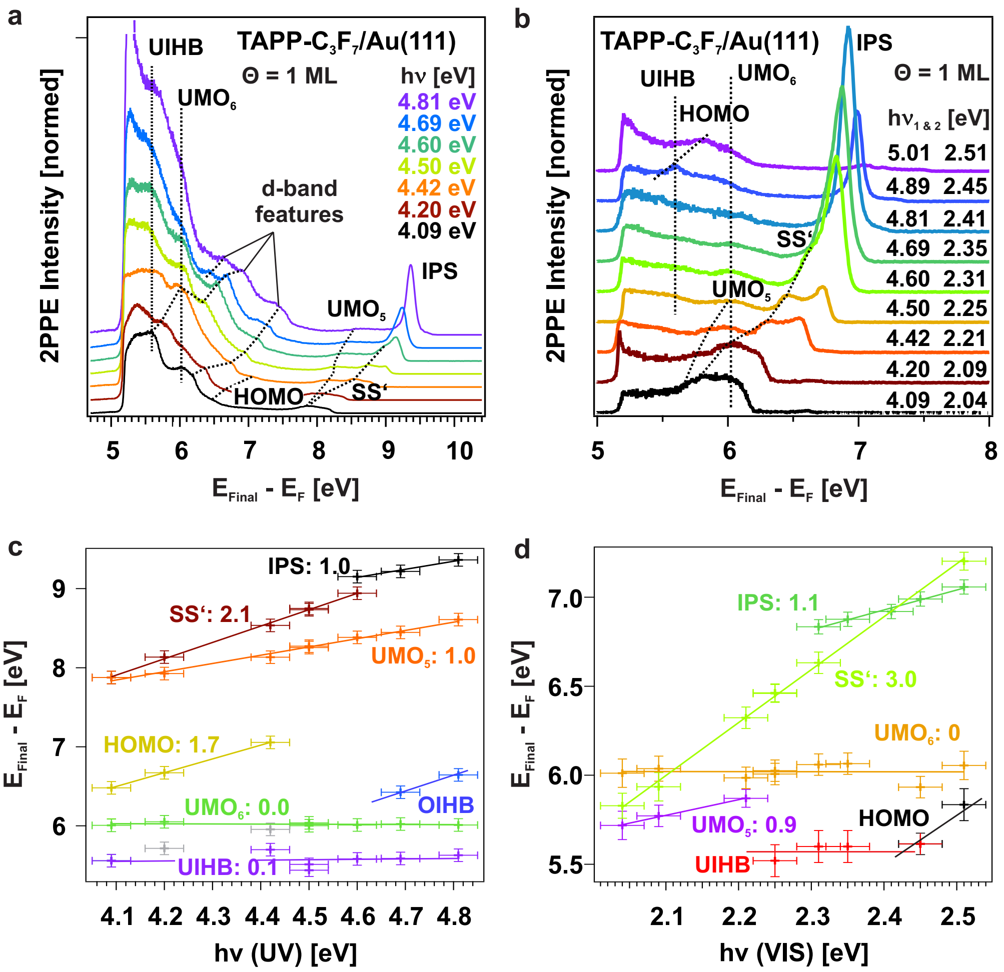

FIG. S5. 2PPE measurements at the TAPP-C_3F_7/Au(111) interface. a) One-color and b) two-color recorded with different photon energies. The peak positions are highlighted by dashed lines. c) and d) Photon-energy dependent peak positions to assign the observed contributions to occupied and unoccupied electronic states.

0.06 eV, $E_{OIHB} = -2.50 \pm 0.06\ eV$, $E_{UMO_4} = 3.74 \pm 0.06\ eV$, $E_{UMO_5} = 6.02 \pm 0.07\ eV$, $E_{UIHB} = 5.57 \pm 0.06\ eV$, and $E_{IPS_{n=1}} = 4.53 \pm 0.07\ eV$.

S5. ADSORPTION PROPERTIES OF TAPP-C_3F_7/AU(111)

STM images of TAPP-C_3F_7 deposited on a Au(111) surface are shown in Fig. S6. The molecules form large self-assembled islands with the molecules flat lying on the surface (Fig. S6a,b). High-resolution images (Fig. S6c) reflect the adsorption structure with the molecules forming a rhombic unit cell with side lengths of a 1.27(5) nm, b=1.28(5) nm, enclosing an

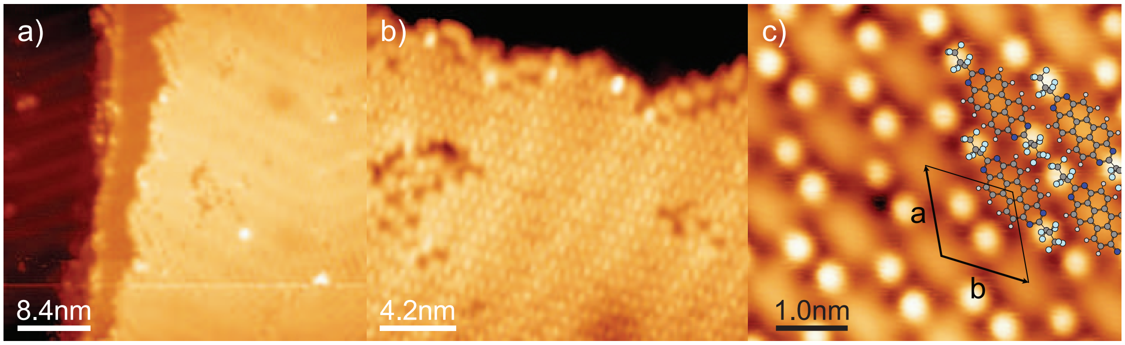

FIG. S6. STM images of TAPP-C_3F_7 on Au(111). a) Overview image: the upper Au terrace contains a large island of densely packed molecules. The herringbone reconstruction is still visible on the molecular island. b) Zoom into a molecular island. c) High resolution STM image. The molecular structure is superimposed to the image to identify individual molecules within the molecular arrangement. The unit cell size is a = 1.27(5)nm × b = 1.28(5)nm with an angle of $\approx 177°$ between a and b, resulting in a unit cell area of $\approx 1.45(5) nm^2$. STM feedback parameters were: (a, b) $V_{bias} = 0.9$ V, $I_{t=110}$ pA, (c) $V_{bias} = 0.56$ V, $I_{t=240}$ pA.

angle of 177°. The bright protrusions at the molecules' terminations are ascribed to the -C_3F_7-groups.
